# Supplementary material for: Rapid whole cell imaging reveals a calcium-APPL1-dynein nexus that regulates cohort trafficking of stimulated EGF receptors
Source: Commun Biol. 2021 Feb 17;4:224. doi: 10.1038/s42003-021-01740-y (PMC7889693; doi:10.1038/s42003-021-01740-y)
Supplement: Supplementary file 2 — Supplementary Information [file 42003_2021_1740_MOESM2_ESM.pdf]

Supplementary Information

RAPID WHOLE CELL IMAGING REVEALS A CALCIUM-APPL1-DYNEIN NEXUS THAT REGULATES COHORT TRAFFICKING OF STIMULATED EGF RECEPTORS

York H M[1,2], Patil, A[1,2], Moorthi U K[1,2], Kaur A[3], Bhowmik A[3], Hyde G J[4], Gandhi H[1,2], Fulcher A[5], Gaus K[3,6] and Arumugam S[1,2,3,6] *

[1]Monash Biomedicine Discovery Institute, Faculty of Medicine, Nursing and Health Sciences, Monash University, Clayton/Melbourne, VIC 3800, Australia
[2]European Molecular Biological Laboratory Australia (EMBL Australia), Monash University, Clayton/ Melbourne, VIC 3800, Australia
[3] Single Molecule Science, University of New South Wales, Sydney, Australia.
[4] Independent scholar, Sydney, Australia
[5] Monas Micro Imaging, Faculty of Medicine, Nursing and Health Sciences, Monash University, Clayton/Melbourne, VIC 3800, Australia
[6]ARC Centre of Excellence in Advanced Molecular Imaging, UNSW, Sydney, Australia

*to whom correspondence should be addressed:
Senthil.arumugam@monash.edu

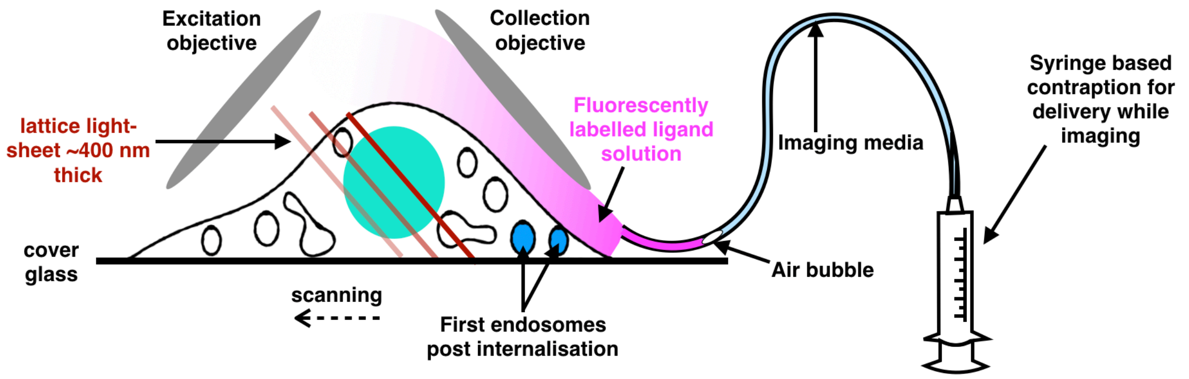

Supplementary figure 1. Cartoon schematic of imaging and cargo delivery arrangement

Cells are excited by a ~400 nm thick multi-Bessel based light sheet and imaged by a perpendicular collection objective during piezo scanning to visualize the entire cell. Cargo is added via a syringe contraption which has an outlet adjacent to the coverslip allowing for direct addition during image acquisition. Given that the volume of the LLSM's sample chamber is 9 ml, sufficient dilution of the injected media was achieved within 2 minutes (supplementary movie 1).

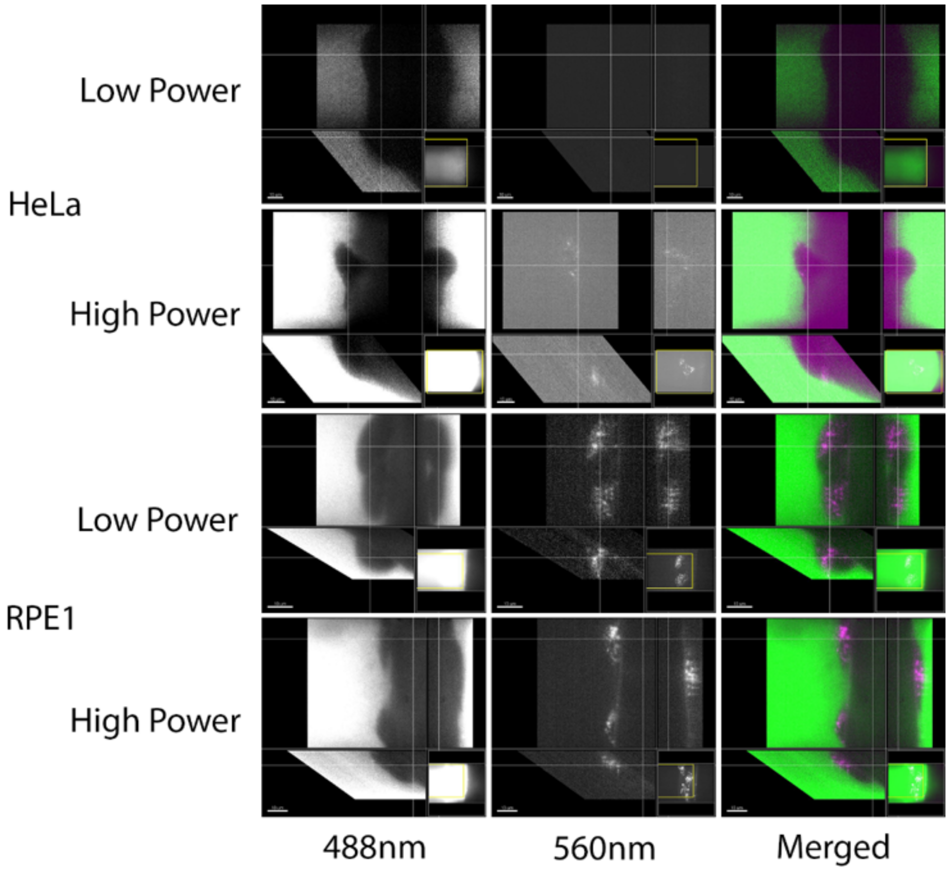

Supplementary figure 2. HeLa cells do not form macropinosomes.
Representative orthogonal views of LLSM imaging of HeLa (top) and RPE1 (bottom) cells following treatment with 100 nM final concentration of Phorbol myristate-13-acetate (PMA) for 2 minutes prior to 100 μg/μL Dextran-fluorescein injection under both low (1%) and high (25%) laser power as indicated. The 488nm channel indicates non-internalized dextran while the 560nm channel indicates internalized dextran as fluorescein is a ratiometric probe for pH that reports on the alkaline endosomes when internalized [1].

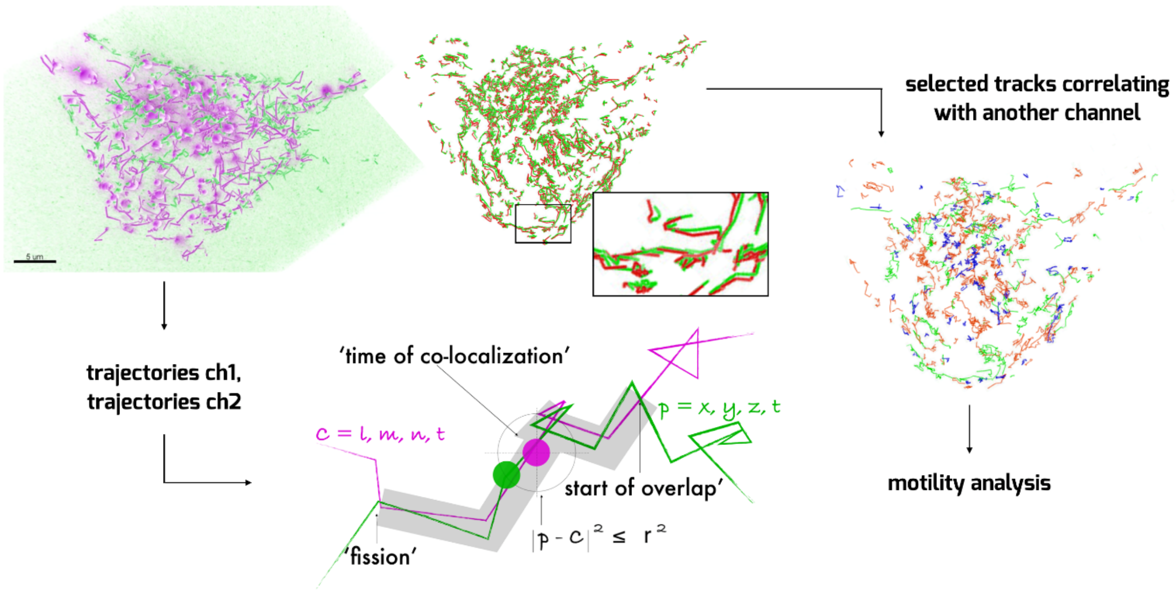

Supplementary figure 3. Co-tracking analysis

Image of detected tracks for two channels (green and magenta). Co-tracking was analyzed by setting the following condition between tracks: $|(p-c)|^2 \leq r^2$ and a time filter of 10 consecutive frames, where p = x, y, z coordinates as a function of time for one channel and c for another. The effective radius of co-localization sphere was set to be 500 nm to account for the sequential imaging and any spatial segregation within a single endosome. The identified tracks that co-track with the second channel can be extracted for further analysis of conditional motility, i.e. motility analysis of tracks showing co-localization of a second channel (Supplementary Movie 3).

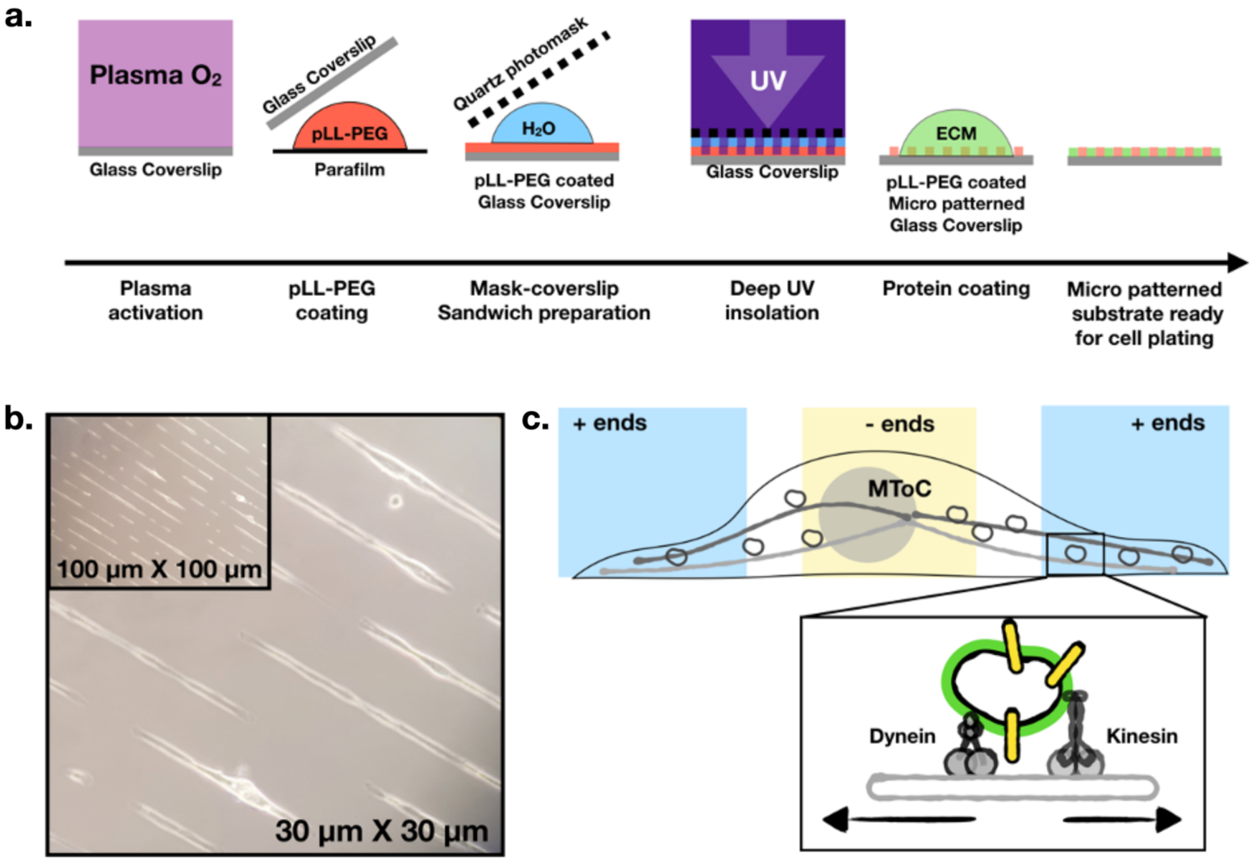

Supplementary figure 4. Micropatterning of coverslips.
(a) Schematic representation of micropatterning process of coverslip (based on [2]). (b) Example image of HeLa cells micropatterned on 5 µm lines, imaged using bright field microscopy at 100x and 30x (insert) magnification. (c) Schematic of microtubule arrangement in an elongated cell highlighting the peripheral plus ends (blue) and peri-nuclear minus ends (yellow) near the microtubule organizing center. Micropatterning cells into thin lines leads to alignment of microtubules within a cell. Rectangular insert corresponds to an APPL1 positive endosome with expected directionality for dynein-based motility and kinesin-based motility with respect to the elongated shape (Supplementary Movie 4).

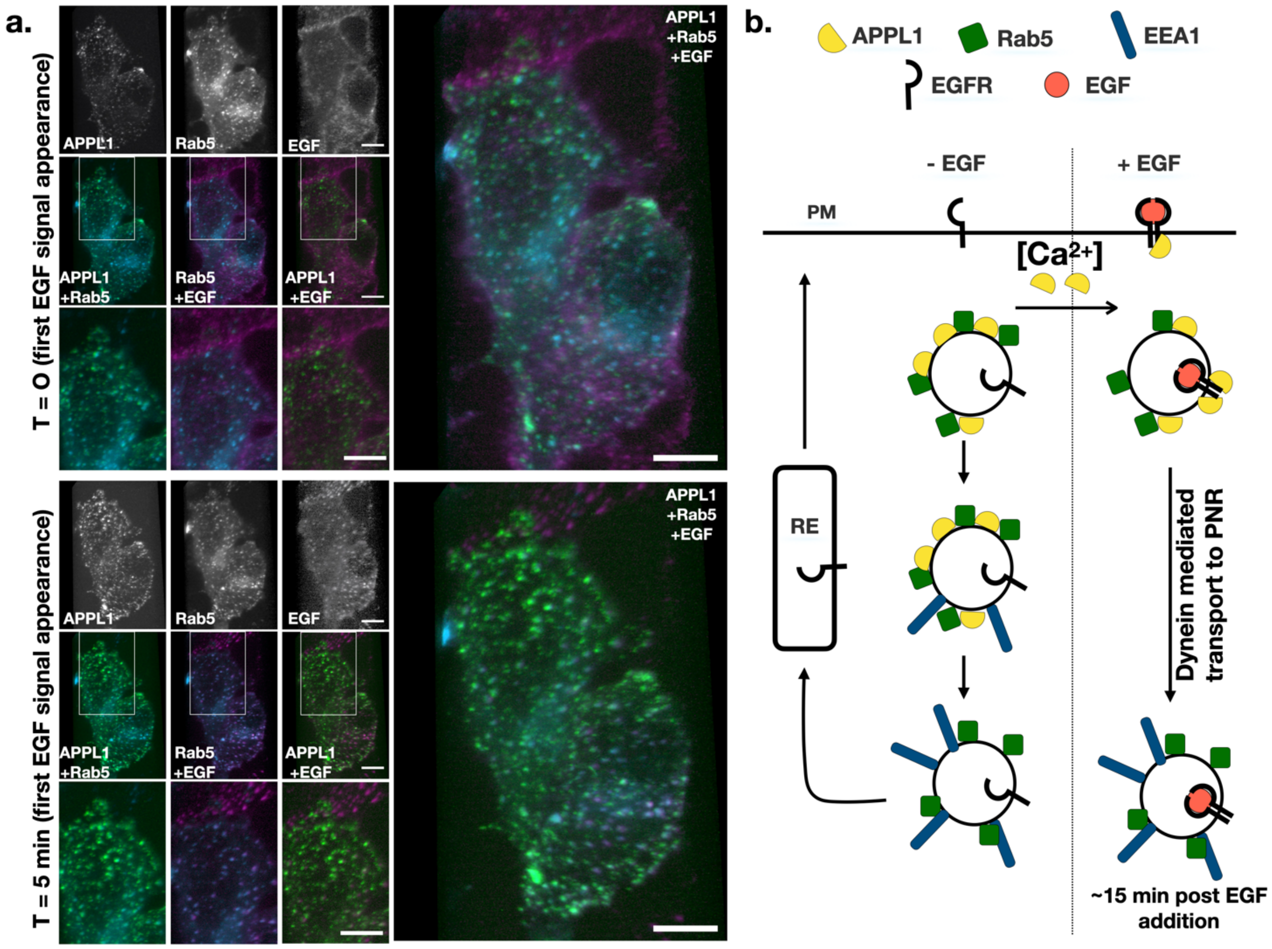

Supplementary figure 5. APPL1 localization pre- and post- EGF stimulation. (a) Top: Live- cell colocalization APPL1-Rab5 in cells immediately after adding EGF. EGF (Magenta in triple colocalized image) is localized at the plasma membrane, while substantial number of endosomes have both APPL1 and Rab5. Bottom: Live- cell colocalization APPL1-Rab5 in cells 5 min after adding EGF, EGF (Magenta) localized to Rab5 and APPL1 double positive structures. (b) A schematic summarizing the overall localization of APPL1 with respect to Rab5 (Supplementary fig. 5a) and EEA1 (Fig.3. in main manuscript). Scale bars = 5 µm.

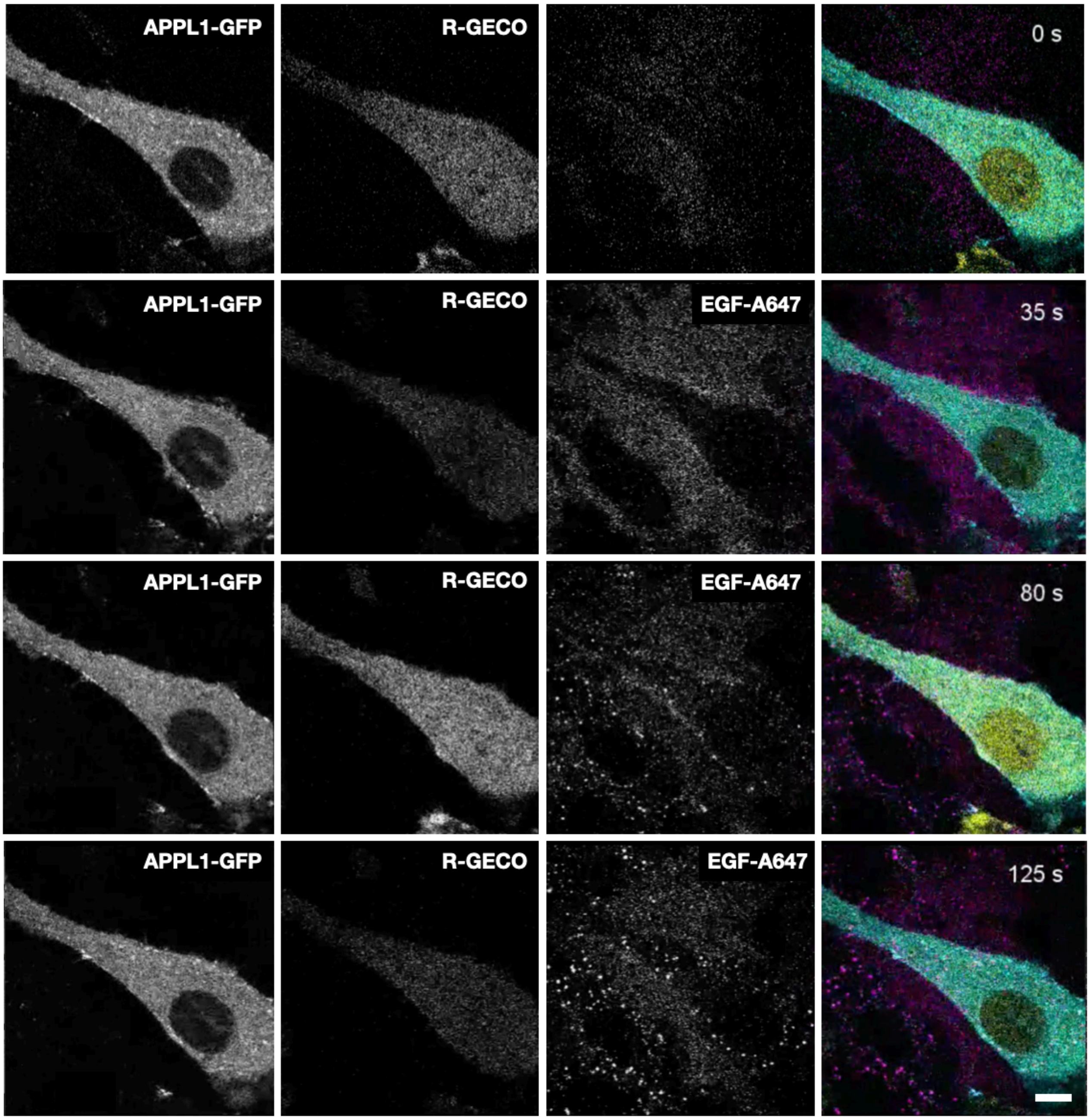

Supplementary figure 6. APPL1-GFP - RGECO - EGFA647 imaging. Representative snapshots of R-GECO signal increase in response to 100 nM EGF stimulation. Calcium increase occurs within 30 to 50 s of EGF binding. Scale bar = 10 μm.

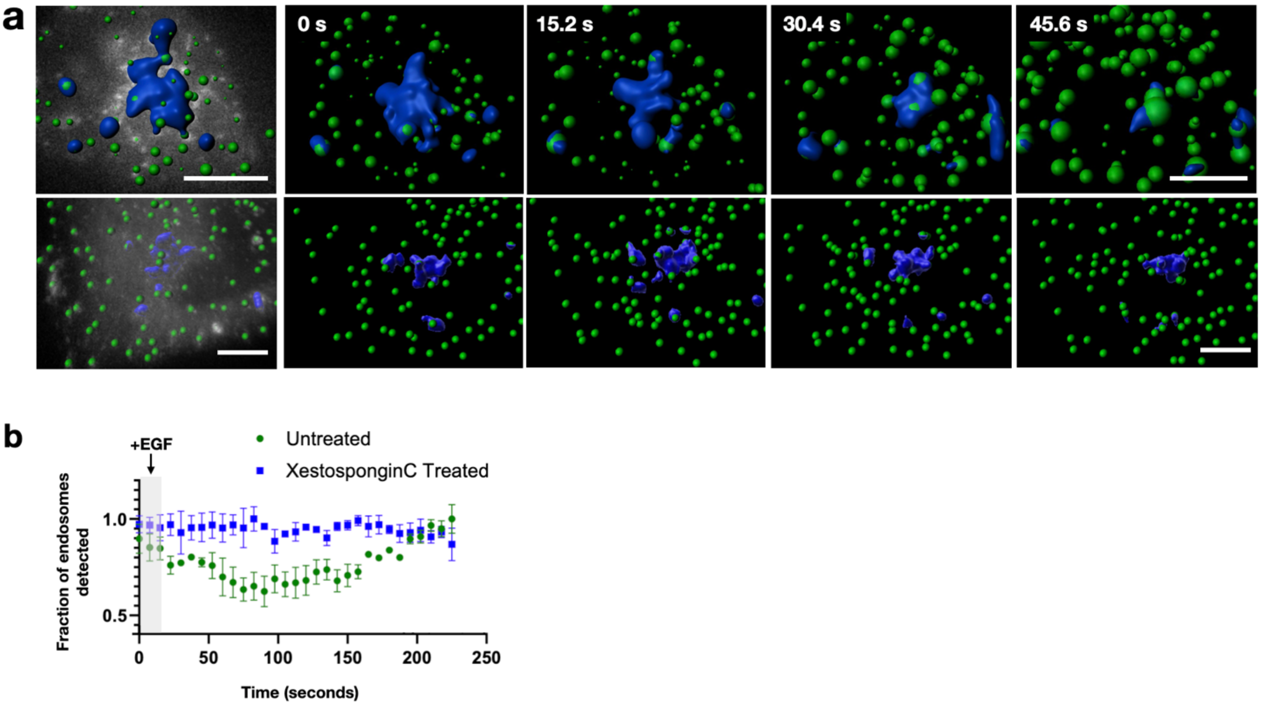

Supplementary Figure 7. Blocking intracellular Ca2+ release impairs transient APPL1 desorption. (a) Example image depicting segmentation of APPL1 EGFP signal in the PNR using Imaris and its loss upon EGF stimulation. Scale bar: 5 µm. **(b)** Plot of number of APPL1 endosomes observed in HeLa APPL1-GFP cells following 100 nM EGF-647 addition for both untreated (green) and 3 uM xestosponginC treated cells (blue) (n=4). Endosomes were detected using the spot detection in Imaris (methods) and normalised for each cell. Error bars show S.D.

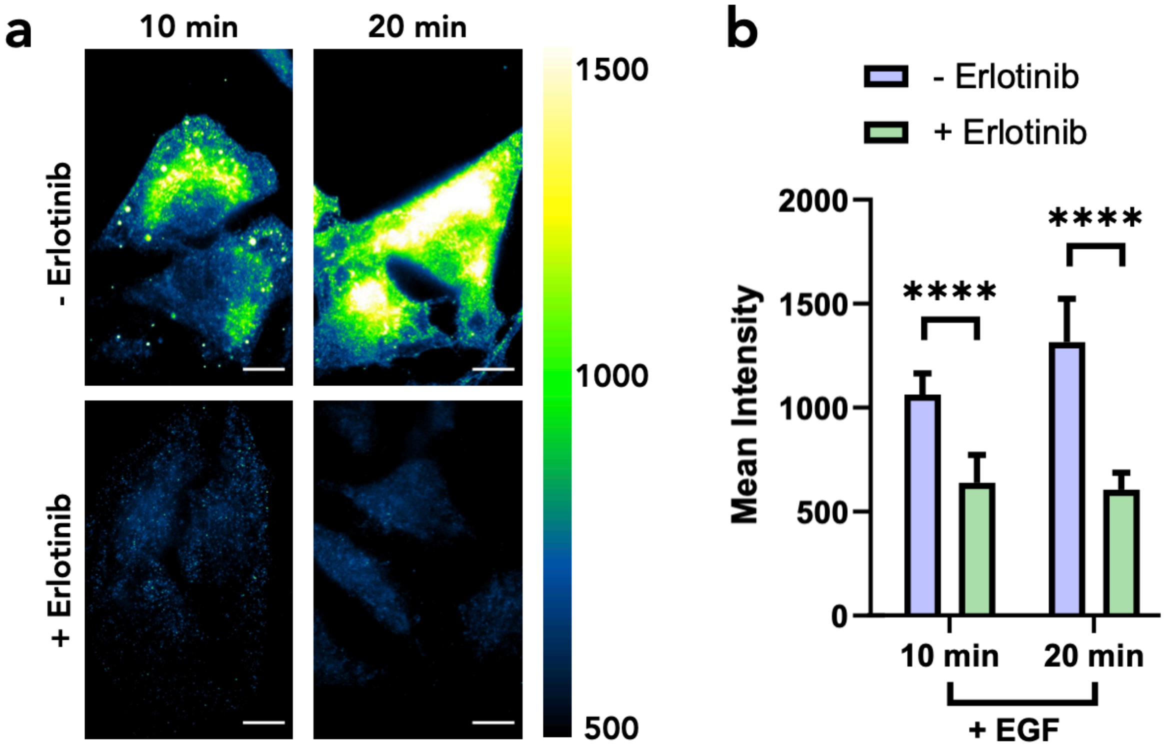

Supplementary figure 8. Phosphorylation inhibition by Erlotinib assayed by anti-phospho-EGFR antibody. (a) (a) Representative intensity colour coded images of showing pEGFR antibody staining in untreated and 10 μM Erlotinib treated cells 10- and 20-minutes post 100 nM EGF addition. Scale bar = 10 μm. (b) Quantification of pEGFR staining in untreated 10 μM Erlotinib treated cells (n = 10). Errors bar show S.D. Significance quantified using an unpaired-T test, **** represents p<0.00001.

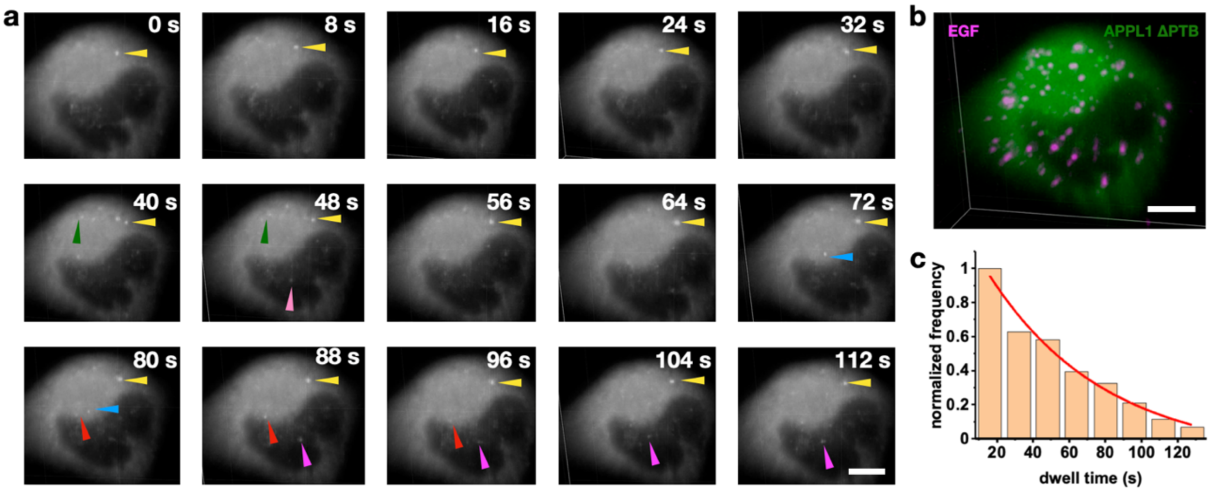

Supplementary figure 9. APPL1 ΔPTB dynamics in cells (a) Representative images of APPL1 ΔPTB that displays transient endosomal localizations. Yellow arrow points at an endosomal stable over 112 s. Magenta arrows point at endosomes with over 24 s of localization, Red arrows point at endosomes with at least 16 s of localization. Green and blue arrows points at endosomes that are transient for at least 8 s. Pink arrow points at an endosome that had APPL1 ΔPTB for only one volume frame (<8s). (b) EGF-647 (magenta) does not localize with APPL1 ΔPTB (Green) (c) Dwell time of punctate APPL1 ΔPTB that reveals transient binding with a $t_{1/2}$ of 64 s. Scale bar = 10 µm.

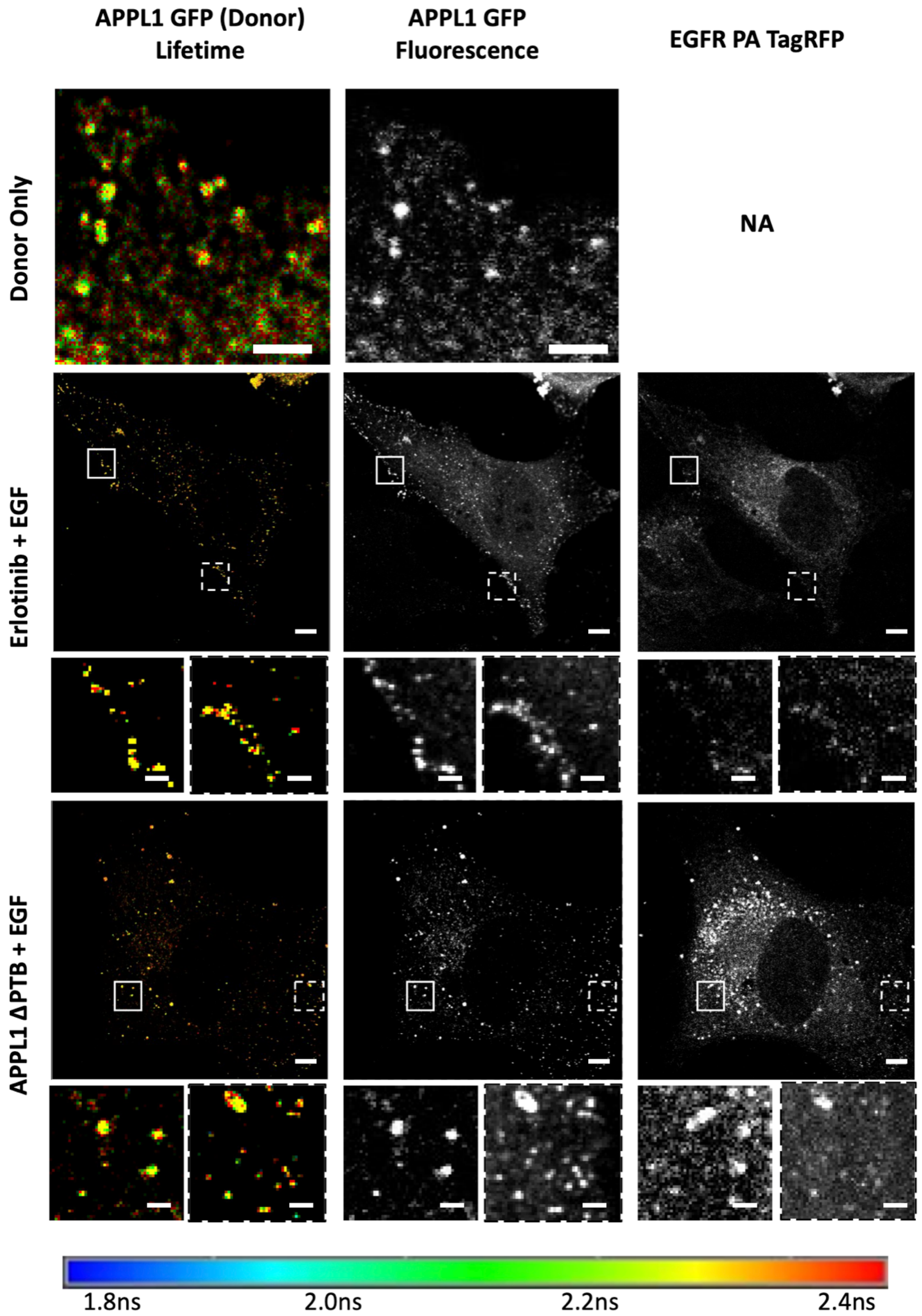

Supplementary figure 10. GFP as a donor in control conditions show no lifetime change. Representative lifetime measurements in donor only (top), Erlotinib treated EGF activated (middle) and cells expressing dominant negative APPL1 lacking the PTB domain (bottom). Scale bar = 5 μm, Scale bars in ROI zooms = 1 μm.

Supplementary References:

[1] Ohkuma, S., and B. Poole. 1978. Fluorescence probe measurement of the intralysosomal pH in living cells and the perturbation of pH by various agents. Proc. Natl. Acad. Sci. USA. 75:3327–3331. doi:10.1073/pnas.75.7.3327

[2] Vignaud *et al.* 2014. Polyacrylamide Hydrogel Micropatterning. Methods in Cell Biology, 120:93-116. doi: 10.1016/B978-0-12-417136-7.00006-9.
