# Supplementary material for: Rapid whole cell imaging reveals a calcium-APPL1-dynein nexus that regulates cohort trafficking of stimulated EGF receptors
Source: Commun Biol. 2021 Feb 17;4:224. doi: 10.1038/s42003-021-01740-y (PMC7889693; doi:10.1038/s42003-021-01740-y)
Supplement: Supplementary file 3 — Description of Additional Supplementary Files [file 42003_2021_1740_MOESM3_ESM.pdf]

Description of Additional Supplementary Files

File Name: Supplementary Data 1

Description: The Source Data behind the graphs

File Name: Supplementary Movie 1

Description: Lattice light-sheet imaging of HeLa transfected with APPL1 EGFP and Alexa 647 labelled GF addition while imaging.

File Name: Supplementary Movie 2

Description: Lattice light-sheet imaging of HeLa transfected with APPL1 EGFP and Alexa 647 labelled Transferrin addition while imaging.

File Name: Supplementary Movie 3

Description: APPL1 movements in EGF stimulated cells, with co-tracking of EGF and APPL1, and retrograde movements resulting in arrival of EGF at Peri-nuclear region.

File Name: Supplementary Movie 4

Description: APPL1 movements in EGF stimulated cells on a micropattern.

File Name: Supplementary Movie 5

Description: Oblique cross section of a single cell demonstrating APPL1 motility characteristics without EGF stimulation.

File Name: Supplementary Movie 6

Description: Oblique cross section of a single cell demonstrating APPL1 motility characteristics upon GF stimulation.

File Name: Supplementary Movie 7

Description: APPL1 motility and EGF motility in cells expressing p150 cc dsRed.

File Name: Supplementary Movie 8

Description: Oblique cross sections of a single cell demonstrating loss of APPL1 signal at PNR upon GF stimulation.

File Name: Supplementary Movie 9

Description: Two cells demonstrating whole-cell redistribution with loss of APPL1 signal at PNR upon GF stimulation.

File Name: Supplementary Movie 10

Description: Segmentation of the PNR to quantify loss of APPL1 signal upon EGF stimulation.

File Name: Supplementary Movie 11

Description: Segmented out visualization of whole cell APPL1 redistribution upon EGF stimulation.

File Name: Supplementary Movie 12

Description: Ionomycin treatment of cells expressing APPL1 GFP and RGECO.
