# Supplementary material for: Rapid whole cell imaging reveals a calcium-APPL1-dynein nexus that regulates cohort trafficking of stimulated EGF receptors
Source: Commun Biol. 2021 Feb 17;4:224. doi: 10.1038/s42003-021-01740-y (PMC7889693; doi:10.1038/s42003-021-01740-y)
Supplement: Supplementary file 17 — Reporting Summary [file 42003_2021_1740_MOESM17_ESM.pdf]

nature research

Corresponding author(s): Senthil Arumugam

Last updated by author(s): Dec 7, 2020

Reporting Summary

Nature Research wishes to improve the reproducibility of the work that we publish. This form provides structure for consistency and transparency in reporting. For further information on Nature Research policies, see our Editorial Policies and the Editorial Policy Checklist.

Statistics

For all statistical analyses, confirm that the following items are present in the figure legend, table legend, main text, or Methods section.

n/a	Confirmed	
☐	☒	The exact sample size (*n*) for each experimental group/condition, given as a discrete number and unit of measurement
☐	☒	A statement on whether measurements were taken from distinct samples or whether the same sample was measured repeatedly
☐	☒	The statistical test(s) used AND whether they are one- or two-sided<br>*Only common tests should be described solely by name; describe more complex techniques in the Methods section.*
☒	☐	A description of all covariates tested
☒	☐	A description of any assumptions or corrections, such as tests of normality and adjustment for multiple comparisons
☐	☒	A full description of the statistical parameters including central tendency (e.g. means) or other basic estimates (e.g. regression coefficient) AND variation (e.g. standard deviation) or associated estimates of uncertainty (e.g. confidence intervals)
☒	☐	For null hypothesis testing, the test statistic (e.g. *F*, *t*, *r*) with confidence intervals, effect sizes, degrees of freedom and *P* value noted<br>*Give P values as exact values whenever suitable.*
☒	☐	For Bayesian analysis, information on the choice of priors and Markov chain Monte Carlo settings
☒	☐	For hierarchical and complex designs, identification of the appropriate level for tests and full reporting of outcomes
☒	☐	Estimates of effect sizes (e.g. Cohen's *d*, Pearson's *r*), indicating how they were calculated

Our web collection on statistics for biologists contains articles on many of the points above.

Software and code

Policy information about availability of computer code

Data collection	Commercial softwares used: Slidebook, LabView, LEICA LAS-X, Oxford Nanoimager.
Data analysis	OriginLab, IMARIS, LabView, Graphpad Prism 8, LEICA LAS-X, custom codes for Centroid and spread estimation, trajectory correlation analysis (available upon request).

For manuscripts utilizing custom algorithms or software that are central to the research but not yet described in published literature, software must be made available to editors and reviewers. We strongly encourage code deposition in a community repository (e.g. GitHub). See the Nature Research guidelines for submitting code & software for further information.

Data

Policy information about availability of data

All manuscripts must include a data availability statement. This statement should provide the following information, where applicable:

- Accession codes, unique identifiers, or web links for publicly available datasets
- A list of figures that have associated raw data
- A description of any restrictions on data availability

The datasets generated during and/or analyzed during the current study are available from the corresponding author on reasonable request.

Field-specific reporting

Life sciences study design

All studies must disclose on these points even when the disclosure is negative.

Sample size	No statistical method was used to predetermine sample size. Sample size were chosen based on statistical evaluation of the data post acquisition and strength of effect observed.
Data exclusions	Data exclusions were not pre-established. In the APPL1 siRNA experiments, 5 cells out of 36 showed EGF trafficking similar to unperturbed conditions, and were excluded from analysis since siRNA may have not been effectively transfected in these cells. Including this data did not have an effect on the results.
Replication	All attempts at replication of data contained in this manuscript were successful. Experiments were performed at least three times unless otherwise noted.
Randomization	No method of randomization is applicable here.
Blinding	Some of the data sets (Motility characteristics analyzed by Abhishek Patil), was given to him as blind sets and results provided by them were then compiled together to prevent human bias during analysis of experiments that were acquired by a different set (Harrison York, Ullhas K Moorthi, Amandeeo Kaur). Other analysis done by Harrison York, Ullhas K Moorthi, and Aditi Bhowmik were not blind as the experiments were conducted by them so it was not possible to analyze these sets blindly.

Reporting for specific materials, systems and methods

We require information from authors about some types of materials, experimental systems and methods used in many studies. Here, indicate whether each material, system or method listed is relevant to your study. If you are not sure if a list item applies to your research, read the appropriate section before selecting a response.

Materials & experimental systems

n/a	Involved in the study	
☒	☐	Antibodies
☐	☒	Eukaryotic cell lines
☒	☐	Palaeontology and archaeology
☒	☐	Animals and other organisms
☒	☐	Human research participants
☒	☐	Clinical data
☒	☐	Dual use research of concern

Methods

n/a	Involved in the study	
☒	☐	ChIP-seq
☒	☐	Flow cytometry
☒	☐	MRI-based neuroimaging

Eukaryotic cell lines

Policy information about cell lines

Cell line source(s)	HeLa cells were purchased from CellBank Australia. HeLa cells expressin Rab5 GFP was a kind gift from Prof Marino Zerial, MPI-CBG, Dresden.
Authentication	The cell lines used were authenticated using CellBank Australia.
Mycoplasma contamination	ALl cell lines tested negative for Mycoplasma contamination.
Commonly misidentified lines (See ICLAC register)	NA
